# Lactation Duration and Long-Term Thyroid Function: A Study among Women with Gestational Diabetes

**DOI:** 10.3390/nu10070938

**Published:** 2018-07-21

**Authors:** Pranati L. Panuganti, Stefanie N. Hinkle, Shristi Rawal, Louise G. Grunnet, Yuan Lin, Aiyi Liu, Anne C. B. Thuesen, Sylvia H. Ley, Sjurdur F. Olesen, Cuilin Zhang

**Affiliations:** 1 Epidemiology Branch, Division of Intramural Population Health Research, Eunice Kennedy Shriver National Institute of Child Health and Human Development, National Institutes of Health, Bethesda, MD 20817, USA; pranati_panuganti@brown.edu (P.L.P.); hinklesn@mail.nih.gov (S.N.H.); shristi.rawal@rutgers.edu (S.R.); linyy@iu.edu (Y.L.); liua@mail.nih.gov (A.L.); 2 The Warren Alpert Medical School of Brown University, Providence, RI 02903, USA; 3 Department of Nutritional Sciences, School of Health Professions, Rutgers University, Newark, NJ 07107, USA; 4 Centre for Fetal Programming, Department of Epidemiology Research, Statens Serum Institut, DK-2300 Copenhagen, Denmark; Louise.Groth.Grunnet@regionh.dk (L.G.G.); SFO@ssi.dk (S.F.O.); 5 Department of Endocrinology, Rigshospitalet University Hospital, DK-2200 Copenhagen, Denmark; acbaunthuesen@gmail.com; 6 The Danish Diabetes Academy, DK-5000 Odense, Denmark; 7 Department of Epidemiology, Richard M. Fairbanks School of Public Health, Indiana University, Indianapolis, IN 46202, USA; 8 Harvard T.H. Chan School of Public Health, Harvard University, Boston, MA 02115, USA; sylvia.ley@channing.harvard.edu; 9 Channing Division of Network Medicine, Harvard Medical School and Brigham and Women’s Hospital, Boston, MA 02115, USA

**Keywords:** GDM, lactation, thyroid, triiodothyronine, thyroxine, thyroid antibodies

## Abstract

Lactation is associated with reduced postpartum weight retention and a lower risk of several cardiometabolic disorders in population-based studies. We examined the association between lactation and long-term thyroid function among women with history of gestational diabetes mellitus (GDM), a high-risk population for subsequent metabolic complications. The study included 550 women who developed GDM in the Danish National Birth Cohort (1996–2002) and followed-up in the Diabetes & Women’s Health Study (2012–2014). We assessed adjusted associations between cumulative lactation duration and concentrations of thyroid stimulating hormone (TSH), free triiodothyronine (fT3), and free thyroxine (fT4) measured at follow-up. Women with longer cumulative lactation duration tended to have higher fT3 levels (adjusted β and 95% confidence interval (CI) for ≥12 months vs. none: 0.19 (0.03–0.36); *p*-trend = 0.05). When restricted to women with a single lifetime pregnancy to control for parity (*n* = 70), women who lactated for >6 months (vs. none) had higher fT3 levels (0.46 pmol/L (0.12–0.80); *p*-trend = 0.02) and a higher fT3:fT4 ratio (0.61 (0.17–1.05); *p*-trend = 0.007). Our findings suggested that a longer duration of lactation may be related to greater serum fT3 levels and fT3:fT4 ratio 9–16 years postpartum among Danish women with a history of GDM. The association was particularly pronounced among women who only had one lifetime pregnancy.

## 1. Introduction

The thyroid gland is involved in several physiological processes, including glucose metabolism, muscle repair, cardiovascular function, and thermogenesis [1]. Thyroid dysfunction may be manifested as clinical, subclinical, or autoimmune, depending on the levels of thyroid hormones (thyroid stimulating hormone (TSH), free triiodothyronine (fT3), and free thyroxine (fT4)) and anti-thyroid antibodies (e.g., thyroperoxidase antibody (anti-TPO), and thyroglobulin antibody (anti-TG)). Women who develop diabetes in pregnancy, or gestational diabetes mellitus (GDM), are particularly subject to a substantially increased risk for cardiometabolic disorders [2]. Given the importance of thyroid hormones in maintaining the function of multiple systems and regulatory pathways related to cardiometabolic functions, the identification of potentially modifiable factors related to thyroid function among GDM women is of important clinical and public health significance. 

Lactation has been associated with several health benefits in women [3]. Foremost, postpartum weight retention is reduced in women who breastfed their children [3]. In addition, women with a longer lifetime duration of lactation are at lower risk for cardiometabolic disorders, including cardiovascular disease, type 2 diabetes mellitus (T2DM), and hypertension [3]. Interestingly, animal data also demonstrate that increasing lactation duration is associated with heightened thyroid activity, resulting in increased levels of biologically active thyroid hormone, triiodothyronine (T3) [4]. Specifically, in bovine models, mRNA transcripts for the enzyme responsible for generating T3 were 6-fold greater with 90 days of lactation compared to levels preceding birth [4]. A link between lactation and thyroid function may lie in the reduction of postpartum weight retention, as weight gain was previously highly associated with TSH and fT4 humans [5]. However, human data are lacking for the influence of lactation on long-term thyroid function among both the general population and women with a history of GDM in particular.

The objective of this study was to examine the association between lactation duration and long-term thyroid function among a high-risk cohort of women with a history of GDM [6,7,8,9,10,11,12,13]. We hypothesize that a longer cumulative lactation duration is associated with improved thyroid function, approximately 9–16 years after the index GDM pregnancy.

## 2. Materials and Methods

### 2.1. Study Design

In the Diabetes & Women’s Health (DWH) Study [14], we followed up women identified as having GDM during the index pregnancy of the Danish National Birth Cohort (DNBC) [15]. The DNBC collected data on maternal demographics, perinatal exposures, and health conditions through four telephone interviews conducted at gestational weeks 12 and 30, and 6 and 18 months postpartum (1996–2002). The DWH Study (2012–2014) enrolled 790 women who had GDM during their index pregnancy, 9–16 years after their enrollment in the DNBC. This analysis was limited to women who completed the DWH Study clinical exam in which biospecimens were collected (*n* = 619) and for whom thyroid markers were available (*n* = 611; 99.0%). Women reporting thyroid disease before the index DNBC pregnancy (*n* = 18; 3.0%) were excluded. We further excluded participants with missing data on breastfeeding duration (*n* = 43; 7.3%) to arrive at the final analytic sample (*n* = 550). All participants provided informed consent. The study was approved by the Regional Scientific Ethical Committee (VEK) of the Capital Region of Denmark (record No. H-4-2013-129).

### 2.2. Ascertainment of Cumulative Lactation History after Index GDM Pregnancy

At the DWH Study follow-up, women retrospectively reported the duration of lactation for each of their pregnancies. The cumulative duration of lactation was calculated by summing the number of lactating months following each birth and was categorized as: none, <6, 6 to <12, or ≥12 months. Because we lacked covariate information for pregnancies before the index DNBC pregnancy, we calculated lifetime lactation duration starting at the index pregnancy and thus, adjusted for parity at the index pregnancy and performed further analyses limited to women who were nulliparous at the index pregnancy.

To assess the validity of recalled lactation duration, we examined the correlation between lactation duration proximally reported for the index DNBC pregnancy at 6 and 18 months postpartum interviews, and the duration recalled for the corresponding pregnancy on the DWH Study follow-up questionnaire 9–16 years postpartum. The correlation was high (*r* = 0.81), and 69.9% of women accurately reported the duration within 1 month. In our analyses, we use the retrospectively reported lactation data as it captured the lactation history over all pregnancies as opposed to only the index pregnancy.

### 2.3. Thyroid Function

At the DWH Study follow-up clinical exam, fasting venous blood samples were collected, processed within 1 h, and stored at −80 °C until being analyzed by a central laboratory following a standardized protocol. All samples were assayed in a single batch by a certified clinical laboratory at the University of Minnesota. Concentrations of thyroid stimulating hormone (TSH) (mIU/L) were measured using a sandwich immunoassay (Roche Diagnostics, Indianapolis, IN, USA). Concentrations of free triiodothyronine (fT3) (pmol/L), free thyroxine (fT4) (ng/dL), anti-TPO (IU/mL), and anti-TG (thyroglobulin antibody; IU/mL) were measured using a competitive immunoassay (Roche Diagnostics, Indianapolis, IN, USA). The fT3:fT4 ratio was calculated by dividing serum concentrations of fT3 (pmol/L) by fT4 (ng/dL). The inter-assay coefficients of variance (CV) were all <6.2% for fT3, fT4, and TSH and <15.1% for the anti-TPO and anti-TG antibodies. 

Thyroid function was assessed based on continuous levels of thyroid hormones (TSH, fT3, fT4, fT3:fT4). Additionally, following the American Thyroid Association guidelines, subclinical hypothyroidism (SCH) was defined as having an elevated TSH (normal range 0.45–4.12 mIU/L) with normal fT4 levels (0.93–1.7 ng/dL) [16]. Subclinical hyperthyroidism was defined as having low to undetectable TSH with normal levels of fT3 (normal range 3.53–6.45 pmol/L) and fT4 (normal range 0.93–1.7 ng/dL) [16,17]. Women with both TSH and fT4 levels within the normal range were classified as euthyroid. Participants who did not meet the criteria for euthyroid, SCH, or subclinical hyperthyroidism were classified as “other”. Lastly, participants were categorized as positive for anti-TPO and anti-TG if their antibody levels were above lab reference ranges (anti-TPO: ≥35 IU/mL; anti-TG: ≥115 IU/mL).

### 2.4. Covariates

Data on potential confounders were available from interview responses at the baseline index DNBC pregnancy. Covariates selected a priori included age (years), socioeconomic status (high or medium level professional, skilled worker, other (student, unskilled, unemployed)), nulliparity (yes, no), smoking during pregnancy (any, none), alcohol during pregnancy (any, none) and pre-pregnancy body mass index (BMI) (<25, 25–29.9, ≥30.0 kg/m^2^) calculated from self-reported height and pre-pregnancy weight. Pre-pregnancy weight was not reported at the time of the index pregnancy for a small sub-set of women (*n* = 41), however, pre-pregnancy weight was reported on the DWH Study follow-up questionnaire. For women who reported a pre-pregnancy weight at both instances the weight was highly correlated (*r* = 0.89) and thus, was used to supplement the missing data when available (*n* = 40).

We considered effect modification by several key variables at the DWH Study follow-up, including T2DM status, age, menopausal status, and long-term weight change since the index pregnancy. T2DM status was classified based on HbA1c levels ≥6.5%, fasting glucose ≥7 mmol/L, 2 h glucose after 75 g oral glucose tolerance test ≥11.1 mmol/L, or self-report of physician diagnosis at follow-up [18]. Type 1 diabetes at follow-up was based on self-report of physician diagnosis. Women self-reported their menopausal status (yes, no). Age was categorized according to the median age in the cohort at follow-up (<45 years vs. ≥45 years). Long-term weight change was calculated as the difference in measured weight at the DWH follow-up and self-reported pre-pregnancy weight at the DNBC index pregnancy and was categorized according to the median level (<4.1 kg vs. ≥4.1 kg). 

### 2.5. Statistical Analysis

Characteristics of study participants were presented overall and by cumulative lactation duration after the index pregnancy. Differences across lactation duration were described as mean (standard deviation, SD) for continuous variables and frequency (%) for categorical variables. Bivariate associations were evaluated using the one-way Analysis of Variance (ANOVA) for continuous variables and the χ^2^ test for categorical variables.

For each lactation category, we used generalized linear models to estimate the unadjusted and adjusted differences in concentrations of TSH, fT3, fT4 and fT3:fT4, by categories of cumulative lactation duration, with no lactation as the reference group. We assessed linear trends (*p-*trend) by using the median for each category of lactation duration as a continuous exposure. We used logistic regression to estimate unadjusted and adjusted odds ratios (ORs) for SCH, anti-TG positivity, and anti-TPO positivity. All multivariable models were adjusted for potential confounders measured at the index pregnancy and included age, socioeconomic status, pre-pregnancy BMI, parity, smoking during pregnancy, and alcohol consumption during pregnancy. An unknown category was used to account for the small proportion of missing covariate data. Due to a small number of women with SCH (*n* = 17), the models were unstable and results for SCH are not reported. 

To ensure that our findings were not biased by pregnancy or lactation characteristics prior to the index DNBC pregnancy and to remove the effect of parity, we repeated our analyses with only women who had one pregnancy overall and thus, were nulliparous at the index pregnancy. By limiting our sample, we also reduced recall bias from women with multiple pregnancies. In this subset of women, lactation was based on only a single pregnancy and was categorized as none, >0 to 6 months, and >6 months. 

To understand whether the observed findings differed across several clinically distinct subgroups of individuals, we tested for a multiplicative interaction with the following characteristics measured at the DWH Study follow-up: age, menopausal status, T2DM status, and long-term weight change. 

Two-tailed *p*-values < 0.05 were considered significant. All statistical analyses were performed using SAS version 9.4 (SAS Institute, Cary, NC, USA).

## 3. Results

Overall, the median cumulative duration of lactation after a median of one (interquartile range 1–2) pregnancy was 9 (interquartile range 4–15) months, including a lactation duration of 0 months for women who reported never lactating (*n* = 62, 11.3%). With an increasing duration of lactation, women tended to be lower in pre-pregnancy BMI, of older age, multiparous, and report no alcohol consumption during the index pregnancy. Thyroid biomarkers for most participants were within the euthyroid range (*n* = 476, 86.6%), while the remainder were classified as SCH (*n* = 19, 3.5%), hyperthyroid (*n* = 7, 1.3%), or other (*n* = 48, 8.7%). Participants in the “other” category did not meet the criteria for SCH, hyperthyroidism, or euthyroid. Several participants were positive for anti-TPO (*n* = 78, 14.2%) and anti-TG (*n* = 69, 12.6%) (Table 1).

There was no significant association between lactation and odds of anti-TG positivity or anti-TPO positivity (Table 2). Table 3 shows the unadjusted and adjusted differences in women’s thyroid marker levels for each category of cumulative lactation, compared to women who never lactated. Women with a longer lactation duration had higher fT3 levels at follow-up, (adjusted β and 95% confidence interval (CI) for ≥12 months vs. none: 0.19 (0.03, 0.36); *p*-trend = 0.05). No significant associations were observed between lactation and concentrations of TSH, fT4, or the fT3:fT4 ratio at follow-up. 

Although there were significant differences in mean levels of several thyroid biomarkers according to long-term weight change and diabetes status at follow-up (Table A1), there was no variation in the lactation duration–thyroid function association according to maternal weight change, diabetes status, or age at follow-up (*p* for interaction >0.05). There was a significant interaction between lactation duration and menopause status for the outcomes of fT3 (*p* = 0.02) and the fT3:fT4 ratio (*p* = 0.03). The results were null among pre-menopausal women, but stronger among post-menopausal women such that a lactation duration of ≥12 months was associated with higher fT3 levels by 0.47 pmol/L compared to women who never lactated (Table A2). 

To tease apart the effect of lactation from the number of pregnancies, we restricted the analyses to women with a single lifetime pregnancy (*n* = 70). Women with a cumulative lactation duration >6 months had significantly higher levels of fT3 (0.46 pmol/L (0.12, 0.80); *p*-trend = 0.02) and a higher fT3:fT4 ratio (0.61 (0.17, 1.05); *p*-trend = 0.007) than women who had never lactated (Table 4). In addition, there was a significant trend (*p* = 0.04) of increasing TSH with increasing lactation duration. 

## 4. Discussion

In the present study following women 9–16 years after GDM pregnancy, a high-risk population for cardiometabolic complications, we examined whether cumulative duration of lactation is associated with long-term thyroid function. Findings from this study suggested that a longer cumulative lactation duration is associated with higher serum fT3 levels and fT3:fT4 ratio approximately 9–16 years after the index pregnancy. The positive association between lactation duration and fT3 levels was more pronounced among nulliparous women at the index pregnancy. While the strengths of these associations are modest, they highlight novel findings, extending the existing literature which demonstrates long-term associations between women’s lifetime duration of lactation and cardiometabolic health to potentially include thyroid function as a novel endpoint [19,20,21]. 

An important feature of our study was distinguishing the effect of cumulative duration of lactation from parity, as these variables are highly correlated. We accomplished this by restricting the study specifically to women who were nulliparous at the index pregnancy and had only a single lifetime pregnancy. The results of our analyses were more robust among this subset, as we removed any residual confounding due to parity. It was meaningful to analyze this subset, as we observed an association between lactation and thyroid biomarker levels even within the shorter lactation periods following only a single birth.

Prior studies have observed that a longer lactation duration is related to lower levels of risk for breast and ovarian cancer, cardiovascular disease, diabetes, hypertension, and hyperlipidemia [3]. There has been limited clinical or epidemiologic research on lactation and long-term maternal thyroid function. Our study adds an intriguing piece to the prior work by linking lactation duration with long-term thyroid function, as we provide suggestive evidence that women who lactated for longer durations of time had higher levels of serum fT3 later in life. Interestingly, we observed that women with even 6 months of lactation, compared to none, had higher levels of fT3 and the fT3:fT4 ratio. 

Several hypotheses may explain the biological mechanisms associating lactation with long-term maternal metabolic health. To support the developing fetus and prepare the mother for lactation, there is an increase in visceral adiposity, circulating lipids, and insulin levels during pregnancy [22]. It is hypothesized that a prolonged duration of lactation helps to rapidly and more completely reverse these metabolic changes by mobilizing the fat stores accumulated during pregnancy [22]. This “reset” to the maternal metabolism may reduce women’s risk for future metabolic diseases, possibly including thyroid dysfunction. Animal models also offer unique insight into the biological pathways linking lactation and thyroid function. In bovine models, the quantity of mRNA transcripts in the mammary gland for iodothyronine deiodinase type II (DIO2), the enzyme responsible for generating T3, the biologically active thyroid hormone, from inactive T4, increased with lactation duration [4]. This observation is consistent with our findings that a longer duration of lactation is associated with greater fT3 levels and fT3:fT4, as fT3 is the form of T3 that is in circulation, and the fT3:fT4 ratio is a proxy for the conversion from T4 to T3. Furthermore, we hypothesized that a potential mechanism for the association between lactation and thyroid function may have been through weight change, yet we did not observe any evidence for this. Lastly, the association between GDM and T2DM has been previously studied and served as the impetus for this research to look beyond cardiometabolic disorders and extend to thyroid metabolism. However, it should be noted that the results were consistent regardless of T2DM status at follow-up. Further research into the mechanisms is warranted.

Although the effect sizes observed in our study do not clinically alter euthyroid status, prior research has demonstrated an association between small changes in thyroid hormone levels and clinical outcomes, even within the euthyroid range. Specifically, the importance of T3 in overall health and metabolic function has been demonstrated among patients with end-stage renal disease, hemodialysis, chronic heart failure, and cerebral infarction who also had clinically low T3 (i.e., low T3 syndrome) and were receiving T3 replacement therapy [23,24,25,26]. Conversely, higher fT3 levels have been associated with several parameters of metabolic syndrome, non-alcoholic fatty liver disease (NAFLD), and insulin resistance among cohorts of healthy euthyroid individuals [27,28,29,30]. The conflicting results from these studies warrant further investigation of lifetime lactation and thyroid disease, beyond clinical biomarkers. Lactation was not associated with thyroid autoimmunity status (i.e., TGO-positivity and TG-positivity); however, the sample size in the current study may have been too small to detect an association. Further research, with a larger sample and longer follow-up time, may be necessary to understand the clinical significance of higher fT3 levels associated with a longer duration of cumulative lactation. 

There are several strengths to our study, including the hybrid study design, combining DNBC pregnancy data with follow-up data from the DWH Study. This study design provided the unique opportunity to follow a large high-risk cohort of women with a history of GDM for over a decade, and to understand their long-term health consequences and modifiable risk factors for specific health outcomes. Additionally, lactation duration, which was reported by women immediately following the index DNBC pregnancy, was highly correlated with women’s reported duration at the DWH Study follow-up 9–16 years later. This also helped to validate women’s recall of lactation duration for the additional births. Our study was further strengthened by consideration of several risk factors for thyroid function reported during the index DNBC pregnancy, including pre-pregnancy BMI, age, socioeconomic status, smoking during pregnancy, and alcohol consumption during pregnancy. Finally, our study is generalizable to Danish women with a history of GDM. While our findings may not be generalizable to all women, women with GDM represent a unique high-risk model for metabolic dysfunction.

Several limitations warrant discussion. First, the cumulative duration of lactation was calculated following the index pregnancy because we did not have detailed lactation data and related covariates before the index pregnancy. However, to account for potential confounding from previous lactation history, we adjusted for the number of pregnancies before the index pregnancy (i.e., parity at the index pregnancy). In addition, we conducted an analysis restricted to women for whom the index pregnancy was their first pregnancy and thus had no prior lactation history. In these analyses, we observed a similar, but stronger positive association between lactation duration and thyroid marker levels. Second, we did not have clear data on how many additional pregnancies were complicated by GDM. Third, we assessed thyroid biomarkers only at a single follow-up and we were unaware of baseline thyroid marker levels before pregnancy or during the interim between the index GDM pregnancy and follow up. While we excluded women with known thyroid disorders prior to pregnancy, we did not have information on the development of, and treatment for, thyroid disease during the period between the index GDM pregnancy and follow up. Fourth, we did not have the power to test for associations between lactation and clinical end points related to thyroid function, such as SCH and other thyroid diseases. Lastly, we did not have data from women on reasons for discontinuing breastfeeding or choosing not to breastfeed, and thus we cannot tease out confounding from other factors (i.e., social factors, milk supply, etc.) that may have contributed to lactation duration.

## 5. Conclusions

In Danish women with a history of GDM, our findings suggest a positive association between a longer duration of lactation and higher levels of thyroid hormone 9–16 years postpartum, even among women with a single lifetime pregnancy. Here we identified lactation as a novel potential modifiable factor for thyroid function, and with replication, these findings may add thyroid function to the wide-array of long-term cardiometabolic outcomes associated with increased lactation duration. As with the findings related to metabolic health, more research is needed to understand the mechanisms behind our findings. A longer follow-up period may help further understanding of the clinical impact of elevated fT3 levels associated with longer lactation duration.

## Figures and Tables

**Table 1:** Participant characteristics overall and by lactation history from the Diabetes & Women’s Health Study.

Characteristics Ascertained at Index Pregnancy (1996–2002)	Overall (*n* = 550)	Cumulative Lactation Duration, Months				*p* *
		None (*n* = 62)	>0 to < 6 (*n* = 106)	6 to < 12 (*n* = 171)	≥12 (*n* = 211)	
Age, years	31.5 (4.5)	31.1 (4.5)	31.8 (4.0)	32.2 (4.6)	30.8 (4.4)	**0.01**
Pre-pregnancy BMI, kg/m^2^						**<0.001**
Unknown	30 (5.5)	6 (9.7)	8 (7.6)	6 (3.5)	10 (4.7)	
<25.0	221 (40.2)	13 (21.0)	30 (28.3)	76 (44.4)	102 (48.3)	
25.0–29.9	143 (26.0)	16 (25.8)	26 (24.5)	45 (26.3)	56 (26.5)	
≥30.0	156 (28.4)	27 (43.6)	42 (39.6)	44 (25.7)	43 (20.4)	
Occupation						**<0.001**
Unknown	49 (8.9)	13 (21.0)	12 (11.3)	9 (5.3)	15 (7.1)	
Professional	254 (46.2)	20 (32.3)	31 (29.3)	82 (48.0)	121 (57.4)	
Skilled worker	152 (27.6)	16 (25.8)	41 (38.7)	47 (27.5)	48 (22.8)	
Other (unskilled worker, unemployed, student)	95 (17.3)	13 (21.0)	22 (20.8)	33 (19.3)	27 (12.8)	
Parity						**<0.001**
Unknown	46 (8.4)	13 (21.0)	11 (10.4)	8 (4.7)	14 (6.6)	
0	200 (36.4)	26 (41.9)	32 (30.2)	41 (24.0)	101 (47.9)	
≥1	304 (55.3)	23 (37.1)	63 (59.4)	122 (71.4)	96 (45.5)	
Drank any alcohol while pregnant						**<0.001**
Unknown	21 (3.8)	8 (12.9)	4 (3.8)	3 (1.8)	6 (2.8)	
No	244 (44.4)	33 (53.2)	42 (39.6)	68 (39.8)	101 (47.9)	
Yes	285 (51.8)	21 (33.9)	60 (56.6)	100 (58.5)	104 (49.3)	
Smoked while pregnant						**0.002**
Unknown	21 (3.8)	8 (12.9)	4 (3.8)	3 (1.8)	6 (2.8)	
No	383 (69.6)	34 (54.8)	69 (65.1)	124 (72.5)	156 (73.9)	
Yes	146 (26.6)	20 (32.3)	33 (31.1)	44 (25.7)	49 (23.2)	
**Characteristics ascertained at follow-up (2012–2014)**						
Age, year	43.6 (4.6)	43 (4.6)	43.9 (4.3)	44.3 (4.7)	43.1 (4.6)	
Weight change, kg						0.36
Unknown	8 (1.5)	2 (3.2)	2 (1.9)	2 (1.2)	2 (1.0)	
<4.1	269 (48.9)	35 (56.5)	52 (49.1)	89 (52.1)	93 (44.1)	
≥4.1	273 (49.6)	25 (40.3)	52 (49.1)	80 (46.8)	116 (55.0)	
Diabetes						**0.02**
Unknown	6 (1.1)	2 (3.2)	2 (1.9)	1 (0.6)	1 (0.5)	
No	388 (70.6)	37 (59.7)	65 (61.3)	120 (70.2)	166 (78.7)	
Type 1	14 (2.6)	1 (1.6)	5 (4.7)	4 (2.3)	4 (1.9)	
Type 2	142 (25.8)	22 (35.5)	34 (32.1)	46 (26.9)	40 (19)	
Post-Menopausal						**0.047**
Unknown	6 (1.1)	2 (3.2)	2 (1.9)	1 (0.6)	1 (0.5)	
No	463 (84.2)	49 (79)	87 (82.1)	137 (80.1)	190 (90.1)	
Yes	81 (14.7)	11 (17.7)	17 (16)	33 (19.3)	20 (9.5)	
TSH (mIU/L)	2.1 (1.5)	2.2 (1.5)	2 (1.3)	2.1 (1.3)	2.2 (1.8)	0.90
fT3 (pmol/L)	4.6 (0.6)	4.5 (0.6)	4.6 (0.7)	4.6 (0.5)	4.7 (0.6)	0.16
fT4 (ng/dL)	1.1 (0.2)	1.1 (0.2)	1.2 (0.2)	1.1 (0.2)	1.2 (0.2)	0.67
fT3:fT4 ratio	4.1 (0.6)	4.1 (0.7)	4 (0.6)	4.1 (0.7)	4.1 (0.6)	0.59
Anti-TPO positive	78 (14.2)	8 (12.9)	13 (12.3)	30 (17.5)	27 (12.8)	0.51
Anti-TG Positive	69 (12.6)	5 (8.1)	9 (8.5)	26 (15.2)	29 (13.7)	0.25

Data are presented as mean (standard deviation; SD) for continuous variables and *n* (%) for categorical variables. * Bolded *p* values represent significant global differences in participant characteristics across lactation duration categories. Abbreviations: BMI, body mass index; TSH, thyroid stimulating hormone; fT3, free triiodothyronine; fT4, free thyroxine; anti-TOP, thyroperoxidase antibody; anti-TG, thyroglobulin antibody.

**Table 2:** Odds of thyroid autoimmunity 9 to 16 years postpartum according to lactation history among women with a history of gestational diabetes, Diabetes & Women’s Health Study.

Outcome	*n*	Unadjusted OR (95% CI)	p *	Adjusted OR ^1^ (95% CI)	p *
TG-positive ^2^					
≥12 months	211	1.82 (0.67, 4.91)	0.23	2.63 (0.74, 9.37)	0.13
6 to <12 months	171	2.04 (0.75, 5.58)	0.16	2.88 (0.80, 10.27)	0.10
<6 months	106	1.06 (0.34, 3.31)	0.92	1.72 (0.44, 6.82)	0.43
None	62	1.00 (reference)		1.00 (reference)	
TPO-positive ^2^					
≥12 months	211	0.99 (0.43, 2.31)	0.98	0.93 (0.36, 2.39)	0.88
6 to <12 months	171	1.44 (0.62, 3.33)	0.40	1.17 (0.46, 2.99)	0.74
<6 months	106	0.94 (0.37, 2.42)	0.90	0.70 (0.24, 2.03)	0.52
None	62	1.00 (reference)		1.00 (reference)	

^1^ Model was adjusted for the following covariates, measured at the index pregnancy: age, pre-pregnancy body mass index (unknown, <25, 25–29.9, ≥30.0 kg/m^2^), occupation (unknown, professional, skilled worker, unskilled/unemployed/student), nulliparity (unknown, yes, no), smoking during pregnancy (unknown, any, none), and alcohol consumption during pregnancy (unknown, any, none). ^2^ TG, thyroglobulin antibody; TPO, thyroid peroxidase antibody. * *p*-values represent significance in the difference in odds of TG- or TPO- positivity for each category of lactation duration compared to no lactation.

**Table 3:** Thyroid biomarker levels 9 to 16 years postpartum according to lactation history among women with a history of gestational diabetes, Diabetes & Women’s Health Study.

Thyroid Biomarker	*n*	Unadjusted β Estimate (95% CI)	*p-Trend* *	Adjusted ^1^ β Estimate (95% CI)	*p-Trend* *
TSH (mIU/L)			0.79		0.87
≥12 months	211	−0.05 (−0.48, 0.38)		−0.03 (−0.47, 0.41)	
6 to <12 months	171	−0.07 (−0.51, 0.37)		−0.03 (−0.49, 0.43)	
<6 months	106	−0.17 (−0.64, 0.31)		−0.13 (−0.61, 0.35)	
None	62	Reference		Reference	
fT3 (pmol/L)			**0.03**		0.05
≥12 months	211	**0.18 (0.01, 0.34)**		**0.19 (0.03, 0.36)**	
6 to <12 months	171	0.10 (−0.07, 0.27)		0.15 (−0.02, 0.32)	
<6 months	106	0.10 (−0.08, 0.28)		0.13 (−0.05, 0.31)	
None	62	Reference		Reference	
fT4 (ng/dL)			0.65		0.55
≥12 months	211	0.03 (−0.02, 0.07)		0.03 (−0.02, 0.07)	
6 to <12 months	171	0.02 (−0.03, 0.07)		0.03 (−0.02, 0.07)	
<6 months	106	0.03 (−0.02, 0.08)		0.04 (−0.01, 0.09)	
None	62	Reference		Reference	
fT3:fT4 ratio			0.24		0.30
≥12 months	211	0.04 −0.14, 0.23)		0.06 (−0.13, 0.25)	
6 to <12 months	171	−0.01 (−0.20, 0.18)		0.03 (−0.16, 0.23)	
<6 months	106	−0.06 (−0.26, 0.14)		−0.05 (−0.25, 0.15)	
None	62	Reference		Reference	

^1^ Model was adjusted for the following covariates, measured at the index pregnancy: age, pre-pregnancy body mass index (unknown, <25, 25–29.9, ≥30.0 kg/m^2^), occupation (unknown, professional, skilled worker, unskilled/unemployed/student), nulliparity (unknown, yes, no), smoking during pregnancy (unknown, any, none), and alcohol during pregnancy (unknown, any, none). * Bolded *p*-trend values represent significant linear trends in thyroid biomarker levels with increasing lactation duration. We assessed *p*-trends by using the median for each category of lactation duration as a continuous exposure.

**Table 4:** Thyroid biomarker levels 9 to 16 years postpartum according to lactation history among women with a history of gestational diabetes who had a single lifetime pregnancy, Diabetes & Women’s Health Study.

Thyroid Biomarker	*n*	Unadjusted β Estimate (95% CI)	p-Trend *	Adjusted ^1^ β Estimate (95% CI)	p-Trend *
TSH (mIU/L)			**0.045**		**0.04**
>6 months	24	0.67 (−0.09, 1.44)		0.78 (−0.03, 1.58)	
>0 to 6 months	26	−0.04 (−0.79, 0.71)		0.14 (−0.65, 0.94)	
None	20	Reference		Reference	
fT3 (pmol/L)			0.17		**0.02**
>6 months	24	0.22 (−0.12, 0.56)		**0.46 (0.12, 0.80)**	
>0 to 6 months	26	0.04 (−0.29, 0.38)		0.27 (−0.06, 0.60)	
None	20	Reference		Reference	
fT4 (ng/dL)			0.07		0.36
>6 months	24	−0.09 (−0.20, 0.01)		−0.05 (−0.15, 0.06)	
>0 to 6 months	26	−0.03 (−0.14, 0.07)		−0.02 (−0.12, 0.09)	
None	20	Reference		Reference	
fT3:fT4 ratio			**0.009**		**0.007**
>6 months	24	**0.55 (0.12, 0.99)**		**0.61 (0.17, 1.05)**	
>0 to 6 months	26	0.12 (−0.31, 0.55)		0.25 (−0.18, 0.69)	
None	20	Reference		Reference	

^1^ Model was adjusted for the following covariates, measured at the index pregnancy: age, pre-pregnancy body mass index (unknown, <25, 25–29.9, ≥30.0 kg/m^2^), occupation (unknown, professional, skilled worker, unskilled/unemployed/student), nulliparity (unknown, yes, no), smoking during pregnancy (unknown, any, none), and alcohol during pregnancy (unknown, any, none). * Bolded *p*-trend values represent significant linear trends in thyroid biomarker levels with increasing lactation duration. We assessed *p*-trends by using the median for each category of lactation duration as a continuous exposure.

## Appendix A

**Table A1:** Thyroid biomarkers according to participant characteristics at follow-up (2012–2014).

Characteristics Ascertained at Follow-Up (2012–2014)	*n*	Thyroid Biomarkers			
		TSH (mIU/L)	fT3 (pmol/L)	fT4 (ng/dL)	fT3:fT4 Ratio
Age					
Unknown	6	1.73 (1.10)	4.0 (1.38)	1.09 (0.41)	3.74 (0.46)
<45	314	2.09 (1.66)	4.65 (0.60)	1.14 (0.15)	4.12 (0.66)
≥45	230	2.20 (1.32)	4.62 (0.52)	1.15 (0.17)	4.07 (0.62)
*p* *		0.41	0.51	0.47	0.32
Weight change, kg					
Unknown	8	2.31 (1.73)	4.19 (1.22)	1.10 (0.35)	3.84 (0.45)
<4.1	265	2.09 (1.31)	4.53 (0.56)	1.17 (0.16)	3.94 (0.62)
≥4.1	277	2.17 (1.70)	4.74 (0.56)	1.13 (0.15)	4.25 (0.64)
*p* *		0.57	**<0.01**	**0.01**	**<0.01**
Diabetes					
Unknown	6	1.73 (1.10)	4.0 (1.38)	1.09 (0.41)	3.74 (0.46)
No	388	2.15 (1.63)	4.61 (0.57)	1.14 (0.15)	4.09 (0.60)
Type 1	14	2.69 (1.44)	4.55 (0.48)	1.04 (0.09)	4.37 (0.43)
Type 2	142	2.04 (1.19)	4.72 (0.58)	1.18 (0.18)	4.09 (0.76)
*p* *		0.29	0.13	**<0.01**	0.28
Post-menopausal					
Unknown	6	1.73 (1.10)	4.0 (1.38)	1.09 (0.41)	3.74 (0.46)
No	463	2.14 (1.55)	4.63 (0.56)	1.14 (0.15)	4.11 (0.65)
Yes	81	2.13 (1.36)	4.70 (0.59)	1.18 (0.19)	4.05 (0.62)
*p* *		0.99	0.29	0.05	0.43

Data are presented as the mean (standard deviation; SD) of each thyroid biomarker (i.e., TSH, fT3, fT4, fT3:fT4), according to categories of participant characteristics (i.e., age, weight change, diabetes status, menopause status). * Bolded *p*-values represent significant global differences in the levels of each thyroid biomarker across non-missing categories of participant characteristics.

**Table A2:** Associations between lactation duration and fT3 and the fT3:fT4 ratio by menopausal status among women with a history of gestational diabetes, Diabetes & Women’s Health Study.

Thyroid Biomarker	Pre-Menopausal Women (*n* = 463)		*p-Trend* *	Post-Menopausal Women (*n* = 81)		*p-Trend* *
	*n*	Adjusted ^1^ β Estimate (95% CI)		*n*	Adjusted ^1^ β Estimate (95% CI)	
fT3 (pmol/L)			0.29			0.22
≥12 months	190	0.10 (−0.08, 0.28)		20	**0.47 (0.04, 0.90)**	
6 to <12 months	137	0.12 (−0.07, 0.03)		33	0.10 (−0.30, 0.50)	
<6 months	87	0.02 (−0.17, 0.22)		17	**0.46 (0.03, 0.90)**	
None	49	Reference		11	Reference	
fT3:fT4 ratio			0.43			0.33
≥12 months	190	0.00 (−0.20, 0.21)		20	0.38 (−0.06, 0.82)	
6 to <12 months	137	0.05 (−0.17, 0.27)		33	−0.12 (−0.53, 0.29)	
<6 months	87	−0.11 (−0.34, 0.12)		17	0.14 (−0.30, 0.59)	
None	49	Reference		11	Reference	

^1^ Model was adjusted for the following covariates, measured at the index pregnancy: age, pre-pregnancy body mass index (unknown, <25, 25–29.9, ≥30.0 kg/m^2^), occupation (unknown, professional, skilled worker, unskilled/unemployed/student), nulliparity (unknown, yes, no), smoking during pregnancy (unknown, any, none), and alcohol during pregnancy (unknown, any, none). * *p*-trend values represent linear trends in thyroid biomarker levels with increasing lactation duration. We assessed *p*-trends by using the median for each category of lactation duration as a continuous exposure.
